# Autoimmune Phenotype Confirmation Intervals and Clinical Indicators in Patients With Latent Autoimmune Diabetes in Adults (LADA) in Southwest China: A Multicenter Retrospective Cross‐Sectional Study

**DOI:** 10.1155/jdr/7481709

**Published:** 2026-09-08

**Authors:** Qiang Zhang, Haojie Zhou, Guniqing Zhu, Ran Li, Weiwei Xu, Ruonan Lin, Yuan Zhao, Hui Li, Xiangfu Gu, Xiaoli Zhu

**Affiliations:** ^1^ School of Nursing, Dali University, Dali, Yunnan, China, dali.edu.cn; ^2^ Department of Cardiovascular Medicine, The Second Affiliated Hospital of Chongqing Medical University, Chongqing, China, cqmu.edu.cn; ^3^ The First School of Clinical Medicine, Southern Medical University, Guangzhou, Guangdong, China, fimmu.com; ^4^ Department of Endocrinology and Metabolism, The Second Affiliated Hospital of Chongqing Medical University, Chongqing, China, cqmu.edu.cn; ^5^ School of Public Health, Dali University, Dali, Yunnan, China, dali.edu.cn; ^6^ Department of Endocrinology and Metabolism, Pu′er People′s Hospital, Pu′er, Yunnan, China; ^7^ Patient Service Center, The First Affiliated Hospital of Dali University, Dali, Yunnan, China, dali.edu.cn; ^8^ Department of Endocrinology, The First Affiliated Hospital of Dali University, Dali, Yunnan, China, dali.edu.cn

**Keywords:** clinical indicators, interval to autoimmune phenotype confirmation, latent autoimmune diabetes in adults (LADA)

## Abstract

**Background:**

Patients with latent autoimmune diabetes in adults (LADA) share overlapping clinical phenotypes with Type 2 diabetes mellitus. Extended autoimmune phenotype confirmation intervals and higher complication risks are observed alongside this phenotypic overlap. Real‐world epidemiological data characterizing this interval and relevant clinical indicators remain scarce for Southwest China. This study is aimed at quantifying the duration of this interval and identifying clinical indicators of prolonged confirmation intervals among hospitalized patients in this understudied region.

**Methods:**

This multicenter retrospective cross‐sectional study recruited consecutive inpatients with confirmed LADA from tertiary hospitals across Southwest China between January 2019 and June 2025. Standardized extraction of baseline demographic, clinical, and laboratory data was performed from electronic medical records. The primary binary outcome was defined as the confirmation interval from initial diabetes diagnosis to autoimmune serological testing, which was dichotomized to distinguish patients with prolonged confirmation intervals. Multivariable binary logistic regression was used to identify clinical indicators showing synchronous coexistence with the intervals. The area under the curve (AUC) and Hosmer–Lemeshow goodness‐of‐fit test were adopted to evaluate the discrimination and calibration of the regression model.

**Results:**

A total of 578 eligible participants were included, among whom 363 (62.8%) presented prolonged confirmation intervals. The median interval was 2 (0, 7) years across the overall cohort and 5 (2, 10) years for the prolonged‐interval group. The multivariate regression model showed satisfactory goodness‐of‐fit. Seven clinical indicators of prolonged confirmation intervals were divided into three categories: demographic and behavioral characteristics including educational level and duration of alcohol consumption; metabolic biomarkers measured at LADA confirmation including inpatient mean blood glucose and 2‐hour C‐peptide levels; advanced diabetic manifestations including diabetic retinopathy, diabetic ketosis, and polypharmacy.

**Conclusions:**

A substantial proportion of hospitalized LADA patients in Southwest China present prolonged autoimmune confirmation intervals. Seven clinical indicators showing stable synchronous coexistence with the intervals were identified. The findings provide region‐specific real‐world evidence characterizing clinical features of patients with such intervals to guide inpatient clinical management. Multicenter prospective studies enrolling community populations are required to examine whether these synchronously coexisting indicators apply to broader patient groups.

## 1. Introduction

Latent autoimmune diabetes in adults (LADA) is a slowly progressive subtype of Type 1 diabetes mellitus (T1DM) characterized by autoimmune‐mediated progressive destruction of pancreatic *β*‐cells [1]. Accurate discrimination of LADA relies on combined assessment of clinical manifestations and islet autoantibody testing [2]. China bears the world′s largest burden of LADA, with approximately 10 million affected individuals [3].

Nevertheless, compared with classic T1DM, LADA presents an insidious onset and indolent disease progression, creating substantial clinical obstacles to timely autoimmune phenotype confirmation in routine care [4, 5]. First, patients with early‐stage LADA retain sufficient endogenous *β*‐cell reserve, exhibiting phenotypes indistinguishable from Type 2 diabetes mellitus (T2DM) and showing noninsulin‐dependent clinical features at initial diagnosis. Second, islet autoantibody testing is not routinely integrated into diagnostic workflows for newly diagnosed diabetes at primary healthcare facilities. Third, single‐point serological testing may yield false‐negative autoantibody results, and repeated serological assessments may be needed to confirm an autoimmune etiology. These clinical barriers coincide with frequent failure to identify LADA patients at initial presentation. Many patients receive empirical T2DM regimens. Disease‐specific interventions are only carried out in follow‐up clinical visits [6].

The prevalence of LADA varies markedly across ethnic and geographic populations, ranging from 2% to 12% among individuals newly diagnosed with T2DM [7]. Approximately 10% of European patients initially labeled as T2DM are subsequently reclassified as LADA [8]. A Chinese multicenter study further reported a 11.7% LADA prevalence among young adults with newly diagnosed T2DM [9]. Suboptimal glycemic control, deferred initiation of insulin and immunomodulatory therapy, and elevated risks of microvascular and macrovascular complications are observed alongside prolonged confirmation intervals, together with heavy clinical and socioeconomic burdens [10, 11].

Existing real‐world epidemiological studies conducted among Southwest Chinese populations rarely explore clinical indicators showing synchronous coexistence with such intervals. Characterizing these indicators can support refined autoimmune phenotype confirmation workflows and individualized clinical management. Accordingly, this multicenter retrospective cross‐sectional study is aimed at quantifying the proportion of hospitalized LADA patients presenting prolonged confirmation intervals and identifying clinical indicators exhibiting synchronous coexistence with these intervals. The findings provide region‐specific clinical evidence describing patients with these intervals to support tailored inpatient follow‐up management.

## 2. Materials and Methods

### 2.1. Study Participants

The multicenter retrospective cross‐sectional study was conducted following the Strengthening the Reporting of Observational Studies in Epidemiology (STROBE) guidelines [12]. Consecutive inpatients with confirmed LADA were enrolled from multiple Grade A tertiary hospitals across Southwest China between January 1, 2019 and June 30, 2025.

All participants met the diagnostic criteria outlined in the 2021 Chinese Expert Consensus on the Diagnosis and Treatment of LADA [3]. Core diagnostic criteria included adult‐onset diabetes at age ≥ 18 years, positive islet autoantibodies indicating autoimmune pancreatic *β*‐cell injury, and absence of insulin dependency for at least 6 months following initial diabetes diagnosis. This domestic consensus was adopted to align screening criteria with epidemiological characteristics of Chinese autoimmune diabetes populations and local clinical practice.

Exclusion criteria were defined as follows: (1) concomitant other systemic autoimmune diseases; (2) incomplete or missing key clinical and laboratory data required for outcome definition and regression modeling.

This study was approved by the Ethics Committee of the First Affiliated Hospital of Dali University and other relevant institutions [approval No. DFY20250520002; 2025 Ethical Review No. 275; Ethics Review 2025‐030<Science>‐01].

### 2.2. Variable Screening Framework of Clinical Indicators for Prolonged Autoimmune Phenotype Confirmation Intervals

Relevant literature was systematically retrieved from six databases, including China National Knowledge Infrastructure (CNKI), Wanfang Database, Chinese Science and Technology Journal Database (VIP), PubMed, Web of Science, and the Cochrane Library. Search terms included “Type 1 Diabetes Mellitus”, “Latent Autoimmune Diabetes in Adults”, “LADA”, and “Diagnostic Delay”. Potential clinical indicators for prolonged autoimmune phenotype confirmation intervals were identified through systematic literature review and real‐world medical record screening [13].

A standardized data extraction form was finalized following two rounds of Delphi expert consultation, with investigators reaching consensus to document all candidate variables. All collected variables were grouped into three analytical domains, including sociodemographic indicators, clinical characteristics, and laboratory biomarkers.

### 2.3. Definition of the Autoimmune Phenotype Confirmation Interval

**Figure 1:**
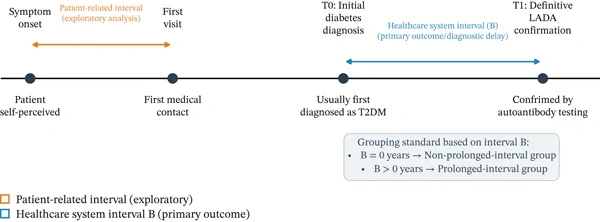
Timeline flow diagram for autoimmune phenotype confirmation intervals.

The full diagnostic timeline displayed in Figure 1 was divided into two sequential time intervals. Interval A corresponded to the patient‐focused interval spanning self‐perceived symptom onset to the first medical contact for diabetes‐related care. Serving as the core time metric of this study, Interval B denoted the institutional timeframe between initial general diabetes diagnosis, most often a tentative T2DM diagnosis, and autoantibody‐verified definitive LADA subtyping. The primary binary outcome variable was generated via dichotomization of Interval B. This metric quantified institutional efficiency of autoimmune phenotype confirmation and supported optimization of routine diabetes diagnostic pathways, which constituted the primary rationale for selecting it as the primary outcome.

Interval B was quantified via calendar‐year differences, calculated as the year of definitive LADA autoantibody confirmation minus the year of initial diabetes diagnosis. This calendar‐based calculation complied with standardized electronic medical record archiving across participating centers, yet this approach lacks month‐level temporal granularity and may induce minor misclassification of this interval metric.

A binary outcome (Interval B > 0 years) served as the primary dependent variable for multivariable logistic regression. This threshold was selected to capture cases where islet autoantibody testing was not performed alongside the initial diabetes diagnosis at first clinical presentation. Multiple alternative definitions of the autoimmune phenotype confirmation interval were adopted in preplanned sensitivity analyses to test the stability of indicators presenting cross‐sectional synchronous coexistence with prolonged intervals.

Participants with an Interval B of 0 years constituted the nonprolonged‐interval group, whereas those with Interval B > 0 years were assigned to the prolonged‐interval group. The untransformed continuous value of Interval B was also retained for regression modeling.

### 2.4. Methods of Data Collection

Two independent data extractors received unified standardized training before formal data extraction. Training covered proficient operation of electronic medical record systems across all participating centers and consistent interpretation of every variable listed in the extraction form. Electronic medical records of all enrolled patients were retrospectively extracted to collect timestamped information on initial diabetes diagnosis and subsequent LADA confirmatory testing.

Five islet autoantibodies were measured in the cohort. Universal autoantibody screening was not available at all participating hospitals, resulting in missing serological data for partial participants. All patients with incomplete autoantibody results were retained in the full cohort and coded as an independent “not available” in all subsequent statistical analyses. This strategy minimized selection bias arising from complete‐case analysis of incomplete samples [14]. All islet autoantibody tests only provided qualitative positive or negative results without quantitative measurement of antibody titers.

### 2.5. Statistical Methods

All raw clinical data underwent double independent entry into Microsoft Excel followed by cross‐verification to eliminate transcription errors. All statistical analyses were conducted using SPSS 25.0 software. The Kolmogorov–Smirnov test was used to assess the normality of continuous variable distributions. Normally distributed continuous variables were summarized as mean ± standard deviation. Nonnormally distributed continuous variables were presented as median (interquartile range). Categorical variables were reported as counts and corresponding percentages.

Univariate between‐group comparisons were performed using independent‐samples *t*‐tests for normally distributed continuous variables and Mann–Whitney *U* tests for nonnormal continuous variables. Chi‐squared tests or Fisher′s exact tests were selected for categorical variable comparisons according to sample cell count assumptions. Between‐group differences in baseline characteristics were evaluated.

A two‐stage screening strategy was implemented to select candidate variables for multivariable logistic regression. Variables with univariate *p* values 0.01 were preliminarily retained as candidates. Clinically meaningful covariates with documented confounding effects from prior publications were also included regardless of univariate *p* values [15]. Variance inflation factors (VIF) were calculated before multivariate modeling to detect multicollinearity. Particular attention was paid to the inherent correlation between total diabetes duration and the autoimmune phenotype confirmation interval, alongside potential interrelatedness between general disease severity covariates and subtyping waiting time. Variables with VIF exceeding 10 were excluded to eliminate severe collinearity, avoiding overlapping disease‐related indicators masking synchronous coexistence signals within this cohort [16]. Backward stepwise multivariable logistic regression was applied to identify clinical indicators presenting cross‐sectional synchronous coexistence with prolonged confirmation intervals. Variable inclusion complied with the events‐per‐variable rule, requiring a minimum of 10 outcome events per variable to mitigate overfitting risks and meet standard analytical specifications [17]. Final model interpretation prioritized clinical plausibility and consistency with published literature rather than statistical screening outputs alone.

Receiver operating characteristic curves, area under the curve values (AUC), and Hosmer–Lemeshow goodness‐of‐fit tests were applied to assess the discriminatory capacity and statistical fitting performance of the regression model. AUC values and Hosmer–Lemeshow tests only validated internal statistical stability of the association results. AUC interpretation criteria were predefined as follows [18]. AUC values < 0.7 represented low discriminative capacity. Values ranging from 0.7 to 0.75 indicated marginal discriminative ability. Values between 0.75 and 0.85 suggested acceptable discriminative validity. Values ≥ 0.85 reflected excellent discriminative performance. The optimal Youden index was calculated to derive corresponding cutoff values, which were only adopted for internal cohort discrimination instead of clinical risk classification. The statistical fitting performance of regression models was considered adequate when the Hosmer–Lemeshow test produced *p* values > 0.05 [19].

Two‐tailed *p* values < 0.05 defined the threshold for statistical significance across all analyses.

### 2.6. Sensitivity Analyses

Preplanned robustness tests were conducted to evaluate whether the cross‐sectional synchronous coexistence observed in primary regression remained stable under different variable definitions of the autoimmune phenotype confirmation interval. The primary binary grouping and calendar‐based time quantification strategy may introduce analytical bias when screening relevant clinical indicators, so three modified outcome specifications were applied for validation [20].

All sensitivity models were designed to incorporate all candidate covariates prespecified for the primary multivariable logistic regression, with three distinct definitions of the autoimmune phenotype confirmation interval set as dependent variables for robustness verification. First, ordinary least squares (OLS) linear regression was run on log‐transformed continuous Interval B, computed as log (years + 1). This transformation retains full temporal data and eliminates arbitrary grouping of interval lengths [21]. Second, logistic regression was constructed around a cutoff of Interval B ≥ 2 years, the median value of the study cohort, which represents a threshold for clinically meaningful prolonged confirmation intervals. Third, an additional logistic regression model adopted the 63rd percentile cutoff of Interval B ≥ 3 years to screen indicators showing cross‐sectional synchronous coexistence among participants with markedly prolonged confirmation intervals [22].

Consistent effect direction and sustained statistical significance (*p* < 0.05) across the primary model and all three sensitivity variants were predefined as the standard for judging analytical robustness. All sensitivity analyses were performed using Python 3.14, statsmodels 0.14.6, and pandas 3.0.1, limited to complete‐case records without missing core modeling variables.

## 3. Results

### 3.1. Occurrence of Prolonged Autoimmune Phenotype Confirmation Intervals Among Hospitalized LADA Patients

A total of 578 patients with LADA were enrolled in this study, consisting of 355 males (61.4%) and 233 females (38.6%). The age of participants ranged from 18 to 84 years, with a median age of 51 (40, 61) years. In terms of educational level, 165 cases (28.5%) had education below junior high school, and 413 patients (71.5%) had junior high school education or above.

Based on the definition of the primary outcome, 363 patients (62.8%) were classified into the prolonged‐interval group, whereas the remaining 215 patients (37.2%) belonged to the nonprolonged‐interval group. The overall median duration of this interval among all participants was 2 (0, 7) years, and the median duration in the prolonged‐interval group was 5 (2, 10) years.

### 3.2. Analysis of Demographic Characteristics Between the Prolonged‐Interval Group and the Nonprolonged‐Interval Group

Baseline comparisons demonstrated no statistically significant between‐group differences in sex, marital status, and ethnicity (all *p* ≥ 0.05), indicating balanced distribution of these demographic variables across the two groups. Statistically significant between‐group differences were observed with respect to age, educational level, and type of medical insurance (all *p* < 0.05). Full demographic data stratified by group are summarized in Table 1.

**Table 1:** Distribution of demographic characteristics between the prolonged‐interval group and the nonprolonged‐interval group.

Demographic characteristics		Prolonged‐interval group (*n* = 363)	Nonprolonged‐interval group (*n* = 215)	Test statistic	*p*
Age [median (IQR), year]		52 (43, 61)	49 (37, 58.5)	−2.537^a^	0.011
Sex (*n*, %)	Male	223 (61.4%)	132 (61.4%)	<0.001^b^	0.993
	Female	140 (38.6%)	83 (38.6%)		
Educational level (*n*, %)	Below junior high school	92 (25.34%)	73 (33.95%)	4.906^b^	0.027
	Junior high school or above	271 (74.66%)	142 (66.05%)		
Marital Status (*n*, %)	Unmarried	29 (8%)	22 (10.2%)	2.981^b^	0.395
	Married	308 (84.8%)	184 (85.6%)		
	Divorced	17 (4.7%)	5 (2.3%)		
	Widowed	9 (2.5%)	4 (1.9%)		
Ethnicity (*n*, %)	Han Chinese	243 (66.9%)	148 (68.8%)	8.437^c^	0.474
	Bai ethnic group	75 (20.7%)	41 (19.1%)		
	Yi ethnic group	25 (6.9%)	15 (7%)		
	Tibetan ethnic group	5 (1.4%)	5 (2.3%)		
	Hui ethnic group	4 (1.1%)	2 (0.9%)		
	Miao ethnic group	3 (0.8%)	2 (0.9%)		
	Tujia ethnic group	5 (1.4%)	0 (0%)		
	Naxi ethnic group	3 (0.8%)	0 (0%)		
	Lisu ethnic group	0 (0%)	1 (0.5%)		
	Dong ethnic group	0 (0%)	1 (0.5%)		
Medical insurance type (*n*, %)	Employee medical insurance	159 (43.8%)	75 (34.9%)	6.194^b^	0.045
	Urban–rural resident medical insurance	197 (54.3%)	131 (60.9%)		
	Self‐payment	7 (1.9%)	9 (4.2%)		

^a^Z‐test.

^b^ *χ* ^2^‐test.

^c^Fisher′s exact test.

### 3.3. Analysis of Clinical Characteristics Between the Prolonged‐Interval Group and the NonProlonged‐Interval Group

Statistically significant between‐group differences were detected for the following clinical characteristics (all *p* < 0.05), including duration of alcohol consumption, duration of the interval to autoimmune phenotype confirmation, age at onset, duration of diabetes, total insulin dosage, preprandial insulin dose, long‐acting insulin dosage, diabetic retinopathy, diabetic vascular complications, insulin injection regimen, history of hypoglycemia, diabetic ketosis, diabetic ketoacidosis, diabetic nephropathy, diabetic neuropathy, polypharmacy, and insulin pump use.

In contrast, no statistically significant between‐group differences were observed for body mass index (BMI), duration of smoking, smoking history, alcohol consumption history, history of hypertension, history of chronic heart disease, hyperosmolar hyperglycemic syndrome, diabetic foot, family history of diabetes, oral hypoglycemic agents, continuous glucose monitoring use, and sleep disorders (all *p* ≥ 0.05). Detailed data on the clinical characteristics of both groups are presented in Table S1.

### 3.4. Analysis of Hematological Test Indicators Between the Prolonged‐Interval Group and the Nonprolonged‐Interval Group

Statistically significant between‐group differences were observed in inpatient mean blood glucose, fasting C‐peptide, 1‐hour C‐peptide, 2‐hour C‐peptide, glomerular filtration rate, triglycerides, and high‐density lipoprotein cholesterol between the two groups (all *p* < 0.05). Although the median values of 1‐ and 2‐hour C‐peptide were comparable across groups, the two cohorts exhibited distinct data distributions and interquartile ranges, which contributed to the overall statistical difference.

In contrast, no statistically significant between‐group differences were detected in postprandial blood glucose fluctuation amplitude, maximum blood glucose fluctuation amplitude, glycated hemoglobin, 0.5‐hour C‐peptide, serum creatinine, total protein, total cholesterol, low‐density lipoprotein cholesterol, hemoglobin, red blood cells, white blood cells, C‐reactive protein, 25‐hydroxyvitamin D, urinary microalbumin, potassium, sodium, calcium, urinary protein, insulin autoantibodies (IAA), tyrosine phosphatase antibodies (IA‐2A), glutamic acid decarboxylase antibodies (GADA), zinc transporter 8 antibodies (ZnT8A), and pancreatic islet cell antibodies (ICA) (all *p* ≥ 0.05). Detailed results are presented in Table S2.

A subset of participants lacked complete laboratory results for the five islet autoantibodies (IAA, IA‐2A, GADA, ZnT8A, and ICA) owing to various reasons. All participants were further stratified into a complete antibody testing subgroup and an incomplete antibody testing subgroup for baseline comparison. No statistically significant intersubgroup differences were found in demographic or clinical baseline features (*p* > 0.05). This finding indicated that incomplete antibody testing did not generate obvious selection bias for measured covariates. Baseline comparison data of the two antibody subgroups are shown in Table S3.

### 3.5. Univariate Analysis of Clinical Indicators for Prolonged Autoimmune Phenotype Confirmation Intervals in LADA Patients

Univariate analysis was performed, with binary prolonged confirmation status defined as the dependent variable. Demographic characteristics, clinical features, and laboratory indicators served as independent variables. Statistically significant associations with prolonged confirmation status were observed for age, medical insurance type, duration of alcohol consumption, educational level, duration of the interval to autoimmune phenotype confirmation, age at onset, duration of diabetes, total insulin dosage, preprandial insulin dose, and long‐acting insulin dosage (all *p* < 0.05), as detailed in Table 2. No statistically significant associations were identified for BMI, duration of smoking, postprandial blood glucose fluctuation amplitude, maximum blood glucose fluctuation amplitude, glycated hemoglobin, 0.5‐hour C‐peptide, serum creatinine, total protein, and total cholesterol (all *p* ≥ 0.05), as detailed in Table S4.

**Table 2:** Univariate analysis of clinical indicators for prolonged autoimmune phenotype confirmation intervals in patients with LADA (*p* < 0.05).

Variables	Test statistic	*p*
Age	6.435^a^	0.011
Medical insurance type	6.194^b^	0.045
Duration of alcohol consumption	4.677^a^	0.031
Educational level	4.906^b^	0.027
Duration of the interval to autoimmune phenotype confirmation	427.567^a^	< 0.001
Age at onset	11.341^a^	0.001
Duration of diabetes	338.611^a^	< 0.001
History of hypoglycemia	6.931^b^	0.008
Diabetic ketosis	11.011^b^	0.001
Diabetic ketoacidosis	7.708^b^	0.005
Diabetic retinopathy	12.750^b^	< 0.001
Diabetic nephropathy	7.220^b^	0.007
Diabetic neuropathy	6.555^b^	0.010
Diabetic vascular complications	4.992^b^	0.025
Polypharmacy	10.099^b^	0.001
Insulin delivery regimen	14.472^c^	0.037
Insulin pump use	4.877^b^	0.027
Number of insulin types used	7.811^c^	0.075
Total insulin dosage	11.691^a^	0.001
Preprandial insulin dose	4.739^a^	0.029
Long‐acting insulin dosage	10.385^a^	0.001
Postprandial blood glucose fluctuation amplitude	3.060^a^	0.080
Inpatient mean blood glucose	4.684^a^	0.030
Fasting C‐peptide	9.019^a^	0.003
1‐hour C‐peptide	3.860^a^	0.049
2‐hour C‐peptide	6.474^a^	0.011
Glomerular filtration rate	8.699^a^	0.003
Triglycerides	5.634^a^	0.018
High‐density lipoprotein cholesterol	20.225^a^	< 0.001

^a^Mann–Whitney *U* test.

^b^ *χ* ^2^‐test.

^c^Fisher′s exact test.

^d^ *t*‐test.

### 3.6. Binary Logistic Regression Analysis of Clinical Indicators for Prolonged Autoimmune Phenotype Confirmation Intervals in LADA Patients

Candidate regression variables were screened and subjected to multicollinearity testing. Retained variables had VIF values ranging from 1.044 to 6.228. All values fell below the standard cutoff of 10, which excluded substantial multicollinearity across independent variables. Complete VIF values are presented in Table S5. Binary logistic regression was performed on screened variables to identify clinical indicators presenting cross‐sectional synchronous coexistence with the target interval outcome.

Binary status of prolonged confirmation intervals (0 = nonprolonged confirmation interval, 1 = prolonged confirmation interval) was designated as the dependent outcome variable. All continuous variables were entered into the model as raw measurements. For categorical variables, “employee medical insurance” was set as the reference group for medical insurance type, “continuous insulin pump infusion” served as the reference group for insulin delivery regimens, and “one type” was defined as the reference group for number of insulin types used. The remaining subgroups of the three categorical indicators were recoded into dummy variables and entered into the regression model together with other covariates. Full coding rules for all independent variables are summarized in Table 3.

**Table 3:** Assignment of independent variables.

Independent variables	Assignment
History of hypoglycemia	1 = No, 2 = Yes
Diabetic ketosis	1 = No, 2 = Yes
Diabetic ketoacidosis	1 = No, 2 = Yes
Diabetic retinopathy	1 = No, 2 = Yes
Diabetic nephropathy	1 = No, 2 = Yes
Diabetic neuropathy	1 = No, 2 = Yes
Diabetic vascular complications	1 = No, 2 = Yes
Polypharmacy	1 = No, 2 = Yes
Educational level	1 = Below junior high school, 2 = Junior high school or above

**Figure 2:**
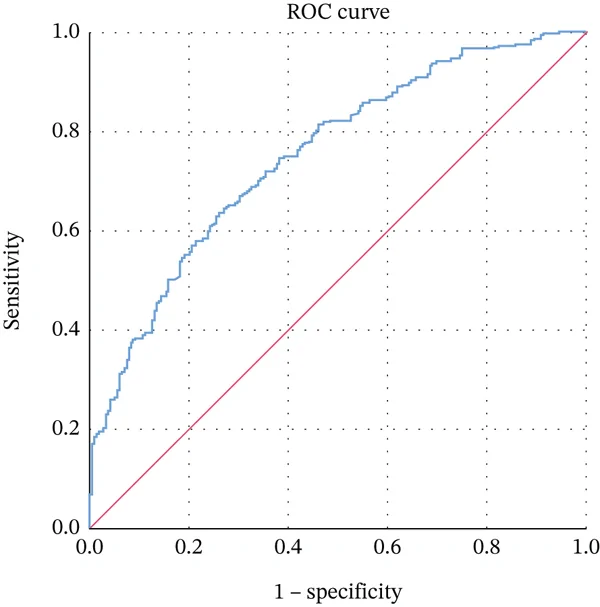
ROC curve for evaluating the overall discriminatory capacity of the logistic regression model. The curve was plotted to evaluate the discriminatory ability of the regression model for distinguishing between the prolonged‐interval and nonprolonged‐interval groups, with the corresponding AUC value calculated to reflect the model fitting performance.

Model discriminatory performance was evaluated via ROC curve following model construction (Figure 2). The AUC was 0.752 (95% CI: 0.712–0.792), which reflected acceptable capacity to distinguish participants across the two groups in the study cohort. The optimal threshold calculated from the maximum Youden index was 0.6412, corresponding to a sensitivity of 63.6% and a specificity of 74.0%. These statistical metrics only supported subgroup differentiation within the present sample. The Hosmer–Lemeshow goodness‐of‐fit test was adopted to examine model fitting performance. The test yielded a *p* value *of* 0.541 (*p* > 0.05), indicating adequate statistical fitting of the regression model.

Regression results demonstrated that duration of alcohol consumption, inpatient mean blood glucose, 2‐hour C‐peptide, diabetic ketosis, diabetic retinopathy, polypharmacy, and educational level exhibited statistically significant cross‐sectional synchronous coexistence with prolonged autoimmune phenotype confirmation intervals (all *p* < 0.05). Detailed results presented in Table 4.

**Table 4:** Multivariable binary logistic regression of clinical indicators for prolonged autoimmune phenotype confirmation intervals in LADA patients (*n* = 578).

Variables	*B*	*SE*	*W* *a* *l* *d* *χ*2	*p*	OR (95% CI)
Duration of alcohol consumption	0.022	0.009	5.796	0.016	1.022 (1.004–1.040)
Inpatient mean blood glucose	0.116	0.046	6.463	0.011	1.124 (1.027–1.229)
2‐hour C‐peptide	‐0.195	0.079	6.012	0.014	0.823 (0.704–0.962)
Diabetic ketosis	‐0.581	0.232	6.286	0.012	0.559 (0.355–0.881)
Diabetic retinopathy	0.727	0.287	6.415	0.011	2.068 (1.179–3.629)
Polypharmacy	0.840	0.264	10.098	0.001	2.315 (1.380–3.886)
Educational level	0.628	0.231	7.359	0.007	1.873 (1.190‐2.948)

### 3.7. Sensitivity Analysis for Alternative Definitions of Autoimmune Phenotype Confirmation Intervals

All robustness assessments were performed on the complete‐case dataset of 578 participants, with effect estimates, 95% confidence intervals and corresponding *p*‐values across all four model specifications summarized in Table S6. The log‐transformed continuous OLS regression model demonstrated satisfactory overall fit (*F* = 11.82, *p* < 0.001, adjusted *R* ^2^ = 0.127). Two logistic regression models using alternative binary cutoffs (≥ 2 years and ≥ 3 years) also reached global statistical significance (both *p* < 0.001), with log‐likelihood values of −364.81 and −366.83, and pseudo R2 values of 0.084 and 0.070, respectively. Across the primary binary logistic model and all three complementary sensitivity models, effect directions for all seven independent covariates remained fully consistent. Five covariates maintained statistical significance (*p* < 0.05) in every analytical specification. Educational level only reached borderline statistical significance in the continuous OLS model (*p* = 0.054) and failed to retain significance at higher interval cutoffs. Inpatient mean blood glucose reached nominal significance solely in the primary logistic model and showed no stable synchronous coexistence across all sensitivity specifications.

## 4. Discussion

This multicenter retrospective cross‐sectional study systematically analyzed clinical data from 578 hospitalized patients with LADA in Southwest China. In this cohort, 62.8% of patients presented prolonged confirmation intervals, with a median interval of 2 years across the entire population and 5 years among participants with prolonged confirmation intervals. These findings reflect a high prevalence of prolonged autoimmune confirmation intervals in routine diabetes care in this region. Notably, the seven identified clinical indicators fall into two distinct groups according to their clinical accessibility at the initial diabetes consultation. The first group comprises late diabetic manifestations that only develop after prolonged disease progression and cannot be obtained upon first diagnosis. The second group encompasses baseline demographic and behavioral characteristics that are easily recorded at the initial clinical encounter. Collectively, the present results fill critical regional epidemiological gaps in clinical characteristics with stable cross‐sectional synchronous coexistence with prolonged confirmation intervals among multiethnic inpatient cohorts, and provide population‐specific clinical evidence to inform refined follow‐up management for affected patients.

### 4.1. High Prevalence of Prolonged Autoimmune Phenotype Confirmation Intervals in Southwest China

The 62.8% proportion of patients with prolonged confirmation intervals observed in the current cohort is substantially higher than previously reported rates in European populations [23], highlighting prominent regional disparities in LADA diagnostic timeliness. Such discrepancies are closely related to heterogeneous local screening protocols and healthcare resource allocation. In European healthcare systems, islet autoantibody testing is standardized and integrated into the initial diagnostic workup for all newly diagnosed diabetes cases, enabling timely identification of autoimmune etiology [24]. In contrast, tertiary and primary healthcare institutions in Southwest China are hindered by nonuniform antibody detection protocols, insufficient screening popularization, and limited accessibility of specialized autoimmune testing [25]. Such systemic healthcare limitations coincide with the high prevalence of prolonged LADA confirmation intervals observed region‐wide.

Multiple institutional, economic, and clinical features coexist with this pattern of prolonged interval formation [26–28]. Autoantibody testing panels are inconsistently available across primary and county hospitals. This situation often accompanies postponed subtype verification. Partial medical insurance coverage generates additional out‐of‐pocket expenses and hinders routine serological screening at initial diabetes visits. Local clinicians tend to prioritize glycemic regulation over autoimmune subtyping upon first diagnosis. Providers only arrange serological testing after patients show poor treatment response or develop advanced diabetic complications. Outpatients and inpatients follow completely different evaluation workflows. Community‐based outpatients only receive fragmented brief follow‐up. Admitted individuals can access full sets of laboratory profiling during hospitalization. All the above contextual features coexist within this tertiary inpatient cohort marked by a high proportion of prolonged confirmation intervals.

### 4.2. Phenotypic Overlap and Ethnic Heterogeneity of Prolonged Autoimmune Phenotype Confirmation Intervals

The phenotypic overlap between LADA and T2DM was prominently reflected in the present cohort [29], with enrolled patients presenting middle‐age onset and normal BMI, representing the typical nonobese diabetic phenotype prevalent in Chinese populations [30]. Diabetes phenotypes vary across ethnic groups, with obesity‐associated T2DM prevailing in Western cohorts and nonobese diabetic presentations more widespread among East Asian populations [31, 32]. Population‐specific clinical traits create prominent overlap between LADA and nonobese T2DM among Chinese patients, meaning the patterns captured in this cohort cannot be extended to Western ethnic populations.

Diagnostic criterion heterogeneity further complicates cross‐population comparison of LADA subtyping outcomes [33–36]. The current study adopted the 18‐year age‐onset threshold based on the 2021 Chinese Expert Consensus, which differs from the 30‐year cutoff recommended by international guidelines. Variations in inclusion criteria may generate inconsistent cohort characteristics across studies, further limiting the extrapolation of Western epidemiological evidence to Chinese LADA populations.

Consistent with the T2DM‐mimicking therapeutic response characteristic of early LADA [37], most patients in this cohort received long‐term T2DM‐oriented treatment prior to formal LADA confirmation [3]. No significant intergroup difference in HbA1c was detected between patients with and without prolonged confirmation intervals. Considering that all participants were tertiary hospital inpatients with generally elevated glycemic levels and complicated disease status, the diminished discriminative ability of routine glycemic markers is clinically plausible. These findings further confirm that isolated glycemic indicators are insufficient to distinguish autoimmune from nonautoimmune diabetes in real‐world clinical scenarios [38].

Beyond phenotypic similarity, inconsistent execution of targeted autoimmune testing represents one widespread clinical practice feature within local healthcare systems [39]. Prolonged confirmation intervals were widely documented even among participants with relatively high educational attainment.

### 4.3. Clinical Indicators for Prolonged Autoimmune Phenotype Confirmation Intervals

Multivariate logistic regression identified seven clinical indicators presenting statistically significant cross‐sectional synchronous coexistence with prolonged confirmation intervals. All these indicators were captured synchronously at the moment of LADA serological confirmation, covering demographic and behavioral characteristics, metabolic markers, and established diabetic complications. All interpretations focus solely on parallel clinical observations recorded within the cohort.

Consistent cross‐sectional synchronous coexistence between core clinical indicators and the autoimmune phenotype confirmation interval was validated across continuous and threshold‐based interval definitions. Uniform directions of effect estimates for all seven covariates and persistent statistical significance of five key clinical indicators across all four model specifications confirm that these synchronous statistical patterns are not artificial artifacts caused by binary grouping or calendar‐year quantification. The fluctuating statistical significance of inpatient mean blood glucose across analytical frameworks further indicates that this inpatient metabolic indicator fails to demonstrate stable synchronous coexistence between itself and prolonged confirmation intervals. Importantly, the identical effect profiles observed in dichotomized and continuous outcome models further rule out the possibility that these synchronous patterns arise merely from confounding linked to overall diabetes duration and progressive disease severity.

#### 4.3.1. Demographic and Behavioral Clinical Indicators

Longer duration of alcohol consumption and lower educational level both showed significant cross‐sectional synchronous coexistence with prolonged confirmation intervals within this cohort. All these data were collected simultaneously at LADA confirmation, and archived medical records lack timestamped data to distinguish event sequence. Prior literature has reported connections between heavy alcohol intake and disturbed glucose metabolism [40]. Detailed drinking history information cannot be retrieved from available medical archives, so the temporal interplay between alcohol exposure and prolonged confirmation intervals cannot be delineated from archived medical records. Future prospective cohorts collecting complete lifestyle data are required to further explore such synchronous statistical patterns.

Lower educational level also presented synchronous coexistence with prolonged confirmation intervals within this cohort. Previous analyses of chronic diabetes cohorts have documented synchronous coexistence between educational level and uptake of specialized serological testing [29]. Most participants in the present cohort held moderate or higher educational levels, while prolonged confirmation intervals were still widely recorded across the sample. Educational stratification and inadequate routine serological screening are two independent contextual observations from this cohort, and existing data cannot quantify their respective weight. These baseline features can be readily collected at initial diabetes visits and provide modest reference value for preliminary risk assessment.

#### 4.3.2. Metabolic Markers at LADA Confirmation

2‐hour C‐peptide levels and inpatient mean blood glucose demonstrated significant synchronous cross‐sectional coexistence with prolonged confirmation intervals. Both metabolic indices were tested at the time of LADA confirmation and only reflect one‐time glycemic and *β*‐cell function snapshots, rather than baseline measurements before initial diabetes diagnosis. 2‐hour C‐peptide is routinely used to evaluate residual pancreatic *β*‐cell function reserve in LADA clinical studies [41]. A portion of participants with impaired postprandial insulin secretion still had relatively short confirmation intervals [42]. Individuals with preserved *β*‐cell reserve show metabolic features matching T2DM. Clinical records document frequent empirical T2DM treatment before formal autoimmune serological assessment for such patients [43]. Combined serological and pancreatic function testing is widely applied for diabetes subtyping in clinical practice.

Elevated in‐hospital mean blood glucose was synchronously observed alongside prolonged confirmation intervals among admitted patients with uncontrolled glycemia. This concurrent metabolic decompensation was only captured at LADA confirmation, making it impossible to clarify whether such glycemic dysregulation preceded autoimmune verification or developed after prolonged waiting for serological assays [44]. Such metabolic abnormalities only emerge after sustained disease progression and cannot be acquired at the time of first diabetes diagnosis.

#### 4.3.3. Diabetic Complications and Advanced Disease Manifestations

Diabetic retinopathy showed synchronous cross‐sectional coexistence with prolonged intervals when measured at LADA confirmation. Chronic hyperglycemia and microvascular complications are frequently recorded together among diabetic populations in prior clinical reports [45]. Clinical records indicate regular glycemic monitoring and specialist referrals are common among individuals with retinopathy [46]. Medical archives do not record clear timelines tracking the onset of retinopathy and serological screening implementation.

Diabetic ketosis displayed inverse synchronous coexistence with prolonged confirmation intervals in this cohort. Ketosis typically develops in advanced LADA accompanied by severe *β*‐cell exhaustion and rarely emerges in early disease stages [41, 47]. For middle‐aged nonobese diabetic patients with ketosis, clinicians should raise vigilance for potential autoimmune diabetes instead of directly diagnosing T2DM.

Polypharmacy also presented significant synchronous coexistence with prolonged confirmation intervals at the time of LADA confirmation. Previous real‐world studies have documented synchronous coexistence between multidrug regimens and complex clinical profiles in older inpatients with diabetes [48]. The current dataset lacks complete medication timeline records to clarify the temporal relationship between polypharmacy and prolonged confirmation intervals. Longitudinal prospective studies with full medication records are needed to resolve this ambiguity. This complication‐related marker develops late in the disease course and lacks utility for early LADA identification at initial consultation.

The incomplete autoantibody testing data available for some participants reflect the real‐world challenge of single‐time‐point serological screening in routine clinical practice. The multivariate regression model exhibited adequate overall goodness‐of‐fit, supporting the reliability of the indicators presenting synchronous coexistence with the target interval outcome.

### 4.4. Study Limitations and Future Perspectives

Several limitations should be acknowledged. First, the primary outcome relied on a binary cutoff of Interval B > 0 years, which groups patients with vastly different subtyping durations. Calendar‐year‐only quantification also lacks monthly precision and may introduce minor measurement misclassification. Predefined sensitivity analyses with continuous and alternative binary interval definitions were performed to validate stable synchronous statistical patterns despite these constraints. Nonetheless, inherent intercorrelation between total diabetes duration and overall disease progression metrics cannot be fully eliminated in this retrospective cross‐sectional cohort. Second, all clinical indicators including retinopathy, ketosis, polypharmacy, inpatient glucose, and C‐peptide were collected cross‐sectionally at LADA confirmation. Retrospective records cannot clarify the chronological order between these manifestations and prolonged confirmation intervals, so all observed synchronous coexistence cannot be interpreted as causal links. Retrospective data also carries inherent information bias even after rigorous quality control. Third, only tertiary hospital inpatients were enrolled, which introduces prominent selection bias. The cohort overrepresents patients with severe glycemic disturbance and multiple diabetic complications, whereas mild community‐based LADA outpatients are excluded. Accordingly, the observed synchronous coexistence cannot be generalized to the full spectrum of routine diabetes patients. Fourth, the adoption of domestic age‐onset diagnostic thresholds creates criterion divergence against international research frameworks, which restricts direct cross‐regional data comparison. Fifth, incomplete autoantibody testing caused by real‐world clinical workflow constraints may introduce residual confounding, even after multivariate adjustment and subgroup analysis. Sixth, this study evaluated the total confirmation interval without further distinguishing intrinsic disease latency from time gaps arising from institutional subtype verification procedures.

Future large‐scale prospective multicenter studies covering both inpatients and community outpatients are required to validate the observed parallel statistical patterns and clarify the chronological sequences of clinical indicators. Such dual‐population cohorts can mitigate selection bias introduced by exclusive inpatient recruitment. Formulating unified domestic diagnostic and screening criteria for Chinese patients with LADA and standardizing hospital islet autoantibody testing workflows can serve as core clinical improvement directions, whereas refined screening frameworks targeting middle‐aged nonobese high‐risk diabetic populations may narrow the scope of prolonged confirmation intervals in relevant clinical groups.

## 5. Conclusions

This multicenter retrospective study characterized the epidemiological profile of autoimmune phenotype confirmation intervals among hospitalized LADA patients in Southwest China, with 62.8% of participants presenting prolonged intervals. Validated multivariate regression analysis identified seven clinical indicators with stable cross‐sectional synchronous coexistence alongside this prolonged timeframe. All indicators were captured simultaneously at LADA serological confirmation, covering demographic‐behavioral features, metabolic parameters, and advanced disease manifestations. Robustness assessments adopting alternative definitions of the confirmation interval further validated the stability of these parallel statistical patterns.

These findings fill regional epidemiological gaps regarding the timing of autoimmune phenotype confirmation for multiethnic Southwest Chinese patients with LADA, and provide real‐world cohort data to support regionally tailored LADA diagnostic protocols. Relevant clinical records furnish neutral references for inpatient LADA care and supplement regional epidemiological evidence to inform targeted adjustments to global LADA diagnostic and management frameworks. Future multicenter prospective cohorts enrolling both inpatients and community‐based outpatients are warranted to validate the observed parallel statistical patterns and broaden the generalizability of the present observations.

## Author Contributions

Qiang Zhang: conceptualization, methodology, software, validation, formal analysis, data curation, writing—review and editing, visualization, project administration, funding acquisition. Haojie Zhou: methodology, software, validation, formal analysis, investigation, resources, data curation, writing—original draft preparation, writing—review and editing, funding acquisition. Guniqing Zhu: methodology, investigation, resources, data curation, writing—original draft preparation. Ran Li: validation, investigation, resources, funding acquisition. Weiwei Xu: investigation, resources. Ruonan Lin: validation, investigation. Yuan Zhao: validation, investigation. Hui Li: investigation, resources. Xiangfu Gu: investigation. Xiaoli Zhu: resources, supervision, project administration. Qiang Zhang, Haojie Zhou, and Guniqing Zhu contributed equally to this article (co‐first authors).

## Funding

This study was supported by the Yunnan Provincial Department of Education Scientific Research Fund Project, 2025Y1186 2026Y1327; Kuanren Talents Program of the Second Affiliated Hospital of Chongqing Medical University.

## Disclosure

All authors have read and approved the final version of the manuscript. Xiaoli Zhu had full access to all of the data in this study and takes complete responsibility for the integrity of the data and the accuracy of the data analysis.

## Ethics Statement

The Ethics Committee of First Affiliated Hospital of Dali University and The Second Affiliated Hospital of Chongqing Medical University approved the study with the approval number: DFY20250520002/Ethics Review No. Two hundred and seventy five of 2025/Ethics Review 2025‐030<Science>‐01. Written informed consent for participation was not required for this study due to its retrospective design, and the study was undertaken in accordance with national legislation and institutional requirements.

## Conflicts of Interest

The authors declare no conflicts of interest.

## Supporting information


**Supporting Information 1** Additional supporting information can be found online in the Supporting Information section. Table S1: Comparison of clinical characteristics between prolonged‐interval group and the nonprolonged‐interval group. Table S2: Comparison of hematological test indicators between the prolonged‐interval group and the nonprolonged‐interval group. Table S3: Baseline characteristics comparison between patients with complete and incomplete five‐islet‐autoantibody detection. Table S4: Univariate analysis of variables showing no statistically significant synchronous coexistence with prolonged autoimmune phenotype confirmation intervals (*p* ≥ 0.05). Table S5: Variance inflation factors of variables included in the final regression model. Table S6: Sensitivity analysis evaluating synchronous coexistence between clinical indicators and prolonged autoimmune phenotype confirmation intervals across alternative model specifications.

## Data Availability

The data that support the findings of this study are available on request from the corresponding author. The data are not publicly available due to privacy or ethical restrictions.
