# Supplementary material for: Autoimmune Phenotype Confirmation Intervals and Clinical Indicators in Patients With Latent Autoimmune Diabetes in Adults (LADA) in Southwest China: A Multicenter Retrospective Cross‐Sectional Study
Source: J Diabetes Res. 2026 Sep 8;2026:7481709. doi: 10.1155/jdr/7481709 (PMC13552359; doi:10.1155/jdr/7481709)
Supplement: Supplementary file 1 [file JDR-2026-7481709-s001.docx]

Table S1 Comparison of clinical characteristics between the prolonged-interval group and the non-prolonged-interval group

| **Clinical characteristics** | | **Prolonged-interval group**  **(*n* = 363)** | **Non-prolonged-interval group**  **(*n* = 215)** | **Test statistic** | ***p* value** |
| --- | --- | --- | --- | --- | --- |
| Body mass index [Median (IQR), kg/m^2^] | | 21.70 (19.60, 23.40) | 21.7 (19.90, 23.70) | -1.153^a^ | 0.249 |
| Duration of smoking [Median (IQR), year] | | 0 (0, 20.00) | 0 (0, 20) | -1.361^a^ | 0.174 |
| Duration of alcohol consumption [Median (IQR), year] | | 0 (0, 10.00) | 0 (0, 0) | -2.163^a^ | 0.031 |
| Duration of the interval to autoimmune phenotype confirmation [Median (IQR), year] | | 5.00 (2.00, 10.00) | 0 (0, 0) | -20.678^a^ | < 0.001 |
| Age at onset [Median (IQR), year] | | 44 .00(34.50, 52.50) | 48.00 (37.00, 58.00) | -3.368^a^ | 0.001 |
| Duration of diabetes [Median (IQR), year] | | 7.00 (3.00, 10.00) | 0 (0, 0) | -18.401^a^ | < 0.001 |
| Total insulin dosage [Median (IQR), IU] | | 32.40 (24.00, 43.00) | 30.00 (21.80, 35.50) | -3.419^a^ | 0.001 |
| Preprandial insulin dose [Median (IQR), IU] | | 20.00 (14.00, 28.00) | 19.00 (12.50, 24.00) | -2.177^a^ | 0.029 |
| Long-acting insulin dosage [Median (IQR), IU] | | 12.40 (9.00, 18.00) | 12.00 (7.00, 14.50) | -3.223^a^ | 0.001 |
| Smoking history (*n*, %) | Y | 177 (48.8%) | 104 (48.4%) | 0.008^b^ | 0.928 |
|  | N | 186 (51.2%) | 111 (51.6%) |  |  |
| Alcohol consumption history (*n*, %) | Y | 106 (29.2%) | 50 (23.3%) | 2.422^b^ | 0.120 |
|  | N | 257 (70.8%) | 165 (76.7%) |  |  |
| History of hypertension (*n*, %) | Y | 81 (22.3%) | 38 (17.7%) | 1.778^b^ | 0.182 |
|  | N | 282 (77.7%) | 177 (82.3%) |  |  |
| History of chronic heart disease (*n*, %) | Y | 32 (8.8%) | 14 (6.5%) | 0.978^b^ | 0.323 |
|  | N | 331 (91.2%) | 201 (93.5%) |  |  |
| Hyperosmolar hyperglycemic syndrome (*n*, %) | Y | 2 (0.6%) | 2 (0.9%) | < 0.001^b^ | 0.990 |
|  | N | 361 (99.4%) | 213 (99.1%) |  |  |
| Diabetic retinopathy (*n*, %) | Y | 84 (23.1%) | 24 (11.2%) | 12.75^b^ | < 0.001 |
|  | N | 279 (76.9%) | 191 (88.8%) |  |  |
| Diabetic vascular complications (*n*, %) | Y | 116 (32.0%) | 50 (23.3%) | 4.992^b^ | 0.025 |
|  | N | 247 (68.0%) | 165 (76.7%) |  |  |
| Diabetic foot (*n*, %) | Y | 5 (1.4%) | 1 (0.5%) | 0.386^b^ | 0.534 |
|  | N | 358 (98.6%) | 214 (99.5%) |  |  |
| Insulin delivery regimen (*n*, %) | None | 9 (2.5%) | 15 (7%) | 14.472^c^ | 0.037 |
|  | Continuous insulin pump infusion | 40 (11%) | 12 (5.6%) |  |  |
|  | Intravenous insulin injection | 2 (0.6%) | 2 (0.9%) |  |  |
|  | Subcutaneous insulin injection ≥ 3 times daily | 260 (71.6%) | 153 (71.2%) |  |  |
|  | Twice-daily subcutaneous insulin injection | 21 (5.8%) | 14 (6.5%) |  |  |
|  | Once-daily subcutaneous insulin injection | 28 (7.7%) | 18 (8.4%) |  |  |
|  | Single subcutaneous insulin injection plus intravenous insulin | 2 (0.6%) | 0 (0%) |  |  |
|  | Multiple subcutaneous insulin injections (≥ 2 times) plus intravenous insulin | 1 (0.3%) | 0 (0%) |  |  |
|  | Weekly insulin formulation | 0 (0%) | 1 (0.5%) |  |  |
| History of hypoglycemia (*n*, %) | Y | 71 (19.6%) | 24 (11.2%) | 6.931^b^ | 0.008 |
|  | N | 292 (80.4%) | 191 (88.8%) |  |  |
| Family history of diabetes (*n*, %) | Y | 73 (20.1%) | 32 (14.9%) | 2.481^b^ | 0.115 |
|  | N | 290 (79.9%) | 183 (85.1%) |  |  |
| Diabetic ketosis (*n*, %) | Y | 117 (32.2%) | 99 (46%) | 11.011^b^ | 0.001 |
|  | N | 246 (67.8%) | 116 (54%) |  |  |
| Diabetic ketoacidosis (*n*, %) | Y | 34 (9.4%) | 37 (17.2%) | 7.708^b^ | 0.005 |
|  | N | 329 (90.6%) | 178 (82.8%) |  |  |
| Diabetic nephropathy (*n*, %) | Y | 43 (11.8%) | 11 (5.1%) | 7.220^b^ | 0.007 |
|  | N | 320 (88.2%) | 204 (94.9%) |  |  |
| Diabetic neuropathy (*n*, %) | Y | 271 (74.7%) | 139 (64.7%) | 6.555^b^ | 0.010 |
|  | N | 92 (25.3%) | 76 (35.3%) |  |  |
| Oral hypoglycemic agents (*n*, %) | Y | 187 (51.5%) | 115 (53.5%) | 0.211^b^ | 0.646 |
|  | N | 176 (48.5%) | 100 (46.5%) |  |  |
| Polypharmacy (*n*, %) | Y | 321 (88.4%) | 169 (78.6%) | 10.099^b^ | 0.001 |
|  | N | 42 (11.6%) | 46 (21.4%) |  |  |
| Continuous glucose monitoring Use (*n*, %) | Y | 8 (2.2%) | 1 (0.5%) | 1.650^b^ | 0.199 |
|  | N | 355 (97.8%) | 214 (99.5%) |  |  |
| Insulin pump use (*n*, %) | Y | 40 (11.0%) | 12 (5.6%) | 4.877^b^ | 0.027 |
|  | N | 323 (89.0%) | 203 (94.4%) |  |  |
| Sleep disorders (*n*, %) | Y | 15 (4.1%) | 3 (1.4%) | 3.352^b^ | 0.067 |
|  | N | 348 (95.9%) | 212 (98.6%) |  |  |

Note: Y: Yes; N: No; a: Z-test; b: χ^2^-test; c: Fisher's exact test

Table S2 Comparison of hematological test indicators between the prolonged-interval group and the non-prolonged-interval group

| **Hematological test indicators** | | **Prolonged-interval group**  **(*n* = 363)** | **Non-prolonged-interval group**  **(*n* = 215)** | **Test statistic** | ***p* value** |
| --- | --- | --- | --- | --- | --- |
| Postprandial blood glucose fluctuation amplitude [Median (IQR), mmol/L] | | 2.17 (1.07, 2.33) | 2.1651 (1.33, 2.9) | -1.749^a^ | 0.080 |
| Maximum blood glucose Fluctuation amplitude [Median (IQR), mmol/L] | | 7.10 (4.80, 10.75) | 6.70 (4.50, 9.90) | -1.541^a^ | 0.123 |
| Inpatient mean blood glucose [Median (IQR), mmol/L] | | 9.62 (8.17, 11.19) | 9.16 (7.52, 10.65) | -2.164^a^ | 0.030 |
| Glycated hemoglobin [Median (IQR), %] | | 10.30 (8.90, 12.70) | 11.06 (8.95, 13.50) | -1.818^a^ | 0.069 |
| Fasting C-peptide [Median (IQR), ng/ml] | | 0.86 (0.30, 0.94) | 0.94 (0.42, 1.35) | -3.003^a^ | 0.003 |
| 0.5-hour C-peptide [Median (IQR), ng/ml] | | 0.74 (0.74, 0.74) | 0.74 (0.61, 1.25) | -1.655^a^ | 0.098 |
| 1-hour C-peptide[Median (IQR), ng/ml] | | 0.88 (0.81, 0.88) | 0.88 (0.71, 1.67) | -1.965^a^ | 0.049 |
| 2-hour C-peptide [Median (IQR), ng/ml] | | 1.38 (1.09, 1.38) | 1.38 (1.04, 2.50) | -2.544^a^ | 0.011 |
| Serum creatinine [Median (IQR), μmol/L] | | 62.00 (52.05, 75.00) | 60.90 (50.00, 71.35) | -1.379^a^ | 0.168 |
| Glomerular filtration rate [Median (IQR), ml/min] | | 104.44 (92.40, 114.43) | 108.80 (96.72, 120.07) | -2.949^a^ | 0.003 |
| Total protein [Median (IQR), g/L] | | 65.14 (60.65, 70.55) | 65.14 (58.90, 70.55) | -0.329^a^ | 0.742 |
| Total cholesterol [Median (IQR), mmol/L] | | 4.50 (3.81, 5.32) | 4.59 (3.72, 5.21) | -0.220^a^ | 0.826 |
| Triglycerides [Median (IQR), mmol/L] | | 1.09 (0.77, 1.62) | 1.29 (0.90, 1.78) | -2.374^a^ | 0.018 |
| Low-density lipoprotein cholesterol [Median (IQR), mmol/L] | | 2.40 (1.91, 2.98) | 2.54 (1.98, 3.05) | -0.913^a^ | 0.361 |
| High-density lipoprotein cholesterol [Median (IQR), mmol/L] | | 1.34 (1.08, 1.62) | 1.17 (0.96, 1.46) | -4.497^a^ | < 0.001 |
| Hemoglobin (g/L, $\bar{\text{x}}\text{±}\text{s}$) | | 137.86±19.88 | 141.08±20.05 | 1.876^d^ | 0.061 |
| Red blood cells [Median (IQR), 10^12^ /L] | | 4.68 (4.24, 5.05) | 4.65 (4.28, 5.12) | -0.881^a^ | 0.378 |
| White blood cells [Median (IQR), 10^9^/L ] | | 6.10 (5.09, 7.55) | 6.30 (5.02, 7.86) | -1.208^a^ | 0.227 |
| C-reactive protein [Median (IQR), mg/L ] | | 13.68 (10.97, 13.68) | 13.68 (7.76, 13.68) | -1.051^a^ | 0.293 |
| 25-Hydroxyvitamin D [Median (IQR), ng/ml] | | 28.00 (28.00, 28.00) | 28.00 (28.00, 28.00) | -1.190^a^ | 0.234 |
| Urinary microalbumin [Median (IQR), mg/L] | | 43.60 (10.50, 81.86) | 28.60 (10.50, 81.86) | -1.228^a^ | 0.219 |
| Potassium (K^+^) [Median (IQR), mmol/L] | | 4.04 (3.80, 4.28) | 4.02 (3.82, 4.23) | -0.690^a^ | 0.490 |
| Sodium (Na^+^) [Median (IQR), mmol/L] | | 139.00 (136.00, 141.00) | 139.00 (136.00, 141.30) | -0.501^a^ | 0.616 |
| Calcium (Ca^2+^) [Median (IQR), mmol/L] | | 2.26 (2.16, 2.37) | 2.24 (2.16, 2.34) | -1.231^a^ | 0.218 |
| Urinary protein (*n*, %) | - | 318 (87.6%) | 188 (87.4%) | 3.789^c^ | 0.401 |
|  | -+ | 20 (5.5%) | 11 (5.1%) |  |  |
|  | 1+ | 19 (5.2%) | 16 (7.4%) |  |  |
|  | 2+ | 3 (0.8%) | 0 (0%) |  |  |
|  | 3+ | 3 (0.8%) | 0 (0%) |  |  |
| Insulin autoantibodies (*n*, %) | Negative | 308 (84.8%) | 192 (89.3%) | 2.294^b^ | 0.130 |
|  | Positive | 55(15.2%) | 23(10.7%) |  |  |
| Tyrosine phosphatase antibodies (*n*, %) | Negative | 103 (28.4%) | 66 (30.7%) | 1.160^b^ | 0.560 |
|  | Positive | 5 (1.4%) | 5 (2.3%) |  |  |
|  | Not Available | 255(70.2%) | 144(67%) |  |  |
| Glutamic acid decarboxylase antibodies (*n*, %) | Negative | 79 (21.8%) | 40 (18.6%) | 0.824^b^ | 0.364 |
|  | Positive | 284(78.2%) | 175(81.4%) |  |  |
| Zinc transporter 8 antibodies (*n*, %) | Negative | 103 (28.4%) | 62 (28.8%) | 4.617^b^ | 0.099 |
|  | Positive | 5 (1.4%) | 9 (4.2%) |  |  |
|  | Not Available | 255(70.2%) | 144(67.0%) |  |  |
| Pancreatic islet cell antibodies (*n*, %) | Negative | 99 (27.3%) | 63 (29.3%) | 1.115^b^ | 0.573 |
|  | Positive | 9 (2.5%) | 8 (3.7%) |  |  |
|  | Not Available | 255 (70.2%) | 144 (67.0%) |  |  |

Table S3 Baseline characteristics comparison between patients with complete and incomplete five-islet-autoantibody detection

| **Variable** | **Test statistic** | ***p* value** | **Variable** | **Test statistic** | ***p* value** |
| --- | --- | --- | --- | --- | --- |
| Age | -5.125^a^ | < 0.001 | High-density lipoprotein cholesterol | -0.214^a^ | 0.831 |
| Body mass index | -1.631^a^ | 0.103 | Hemoglobin | -3.810^a^ | < 0.001 |
| Duration of smoking | -0.996^a^ | 0.319 | Red blood cells | -2.411^a^ | 0.016 |
| Duration of alcohol consumption | -2.041^a^ | 0.041 | White blood cells | -0.947^a^ | 0.344 |
| Duration of the interval to autoimmune phenotype confirmation | -1.947^a^ | 0.051 | C-reactive protein | -4.904^a^ | < 0.001 |
| Age at onset | -2.836^a^ | 0.005 | 25-Hydroxyvitamin D | -6.172^a^ | < 0.001 |
| duration of diabetes | -4.457^a^ | < 0.001 | Urinary microalbumin | -6.851^a^ | < 0.001 |
| Total insulin dosage | -2.829^a^ | 0.005 | Potassium (K^+^) | -1.729^a^ | 0.084 |
| Preprandial insulin dose | -3.678^a^ | < 0.001 | Sodium (Na^+^) | -4.714^a^ | < 0.001 |
| Long-acting insulin dosage | -0.947^a^ | 0.344 | Calcium (Ca^2+^) | -2.423^a^ | 0.015 |
| Postprandial blood glucose fluctuation amplitude | -1.334^a^ | 0.182 | Sex | < 0.001^b^ | 0.991 |
| Maximum blood glucose Fluctuation amplitude | -1.932^a^ | 0.053 | Marital Status | 7.047^b^ | 0.070 |
| Inpatient mean blood glucose | -0.307^a^ | 0.759 | Ethnicity | 69.136^b^ | < 0.001 |
| Glycated hemoglobin | -6.108^a^ | < 0.001 | Medical insurance type | 84.333^b^ | < 0.001 |
| Fasting C-peptide | -5.046^a^ | < 0.001 | Smoking history | 0.127^b^ | 0.722 |
| 0.5-hour C-peptide | -4.711^a^ | < 0.001 | Alcohol consumption history | 11.438^b^ | 0.001 |
| 1-hour C-peptide | -4.994^a^ | < 0.001 | History of hypoglycemia | 0.119^b^ | 0.730 |
| 2-hour C-peptide | -3.822^a^ | < 0.001 | Family history of diabetes | 5.982^b^ | 0.014 |
| Serum creatinine | -2.425^a^ | 0.015 | History of hypertension | 0.001^b^ | 0.974 |
| Glomerular filtration rate | -4.046^a^ | < 0.001 | History of chronic heart disease | 1.992^b^ | 0.158 |
| Total protein | -1.196^a^ | 0.845 | Diabetic ketosis | 21.797^b^ | < 0.001 |
| Total cholesterol | -0.419^a^ | 0.675 | Diabetic ketoacidosis | 0.681^b^ | 0.409 |
| Triglycerides | -0.737^a^ | 0.461 | Hyperosmolar hyperglycemic syndrome | < 0.001^b^ | 1.000 |
| Low-density lipoprotein cholesterol | -0.463^a^ | 0.663 | Diabetic retinopathy | 1.104^b^ | 0.293 |
| Diabetic vascular complications | 6.268^b^ | 0.012 | Diabetic nephropathy | 0.156^b^ | 0.693 |
| Diabetic foot | < 0.001^b^ | 1.000 | Diabetic neuropathy | 4.773^b^ | 0.029 |
| Sleep disorders | 5.613^b^ | 0.018 | Insulin pump use | 10.091^b^ | 0.001 |
| Polypharmacy | 0.191^b^ | 0.662 | Urinary protein | 12.638^b^ | 0.013 |
| Oral hypoglycemic agents | 0.391^b^ | 0.532 | Glutamic acid decarboxylase antibodies | 124.477^b^ | < 0.001 |
| Insulin delivery regimen | 32.258^b^ | < 0.001 | Zinc transporter 8 antibodies | 578.000^b^ | < 0.001 |
| Continuous glucose monitoring Use | 2.762^b^ | 0.097 | Insulin autoantibodies | 1.843^b^ | 0.175 |
| Number of insulin types used | 28.932^b^ | < 0.001 | Tyrosine phosphatase antibodies | 578.000^b^ | < 0.001 |
| Educational level | 3.144^b^ | 0.076 | Pancreatic islet cell antibodies | 578.000^b^ | < 0.001 |
| Total Number of Complications | 31.081^b^ | < 0.001 |  |  |  |

Table S4 Univariate analysis of variables showing no statistically significant synchronous coexistence with prolonged autoimmune phenotype confirmation intervals (*p* ≥ 0.05)

| **Variables** | **Test Statistic** | ***p* Value** | **Variables** | **Test Statistic** | ***p* Value** |
| --- | --- | --- | --- | --- | --- |
| Body Mass Index | 1.329^a^ | 0.249 | Sex | < 0.001^b^ | 0.993 |
| Duration of Smoking | 1.852^a^ | 0.174 | Marital Status | 2.981^b^ | 0.395 |
| Maximum Blood Glucose Fluctuation Amplitude | 2.375^a^ | 0.123 | Ethnicity | 8.437^c^ | 0.474 |
| Glycated Hemoglobin | 3.307^a^ | 0.069 | Smoking History | 0.008^b^ | 0.928 |
| 0.5-Hour C-Peptide | 2.740^a^ | 0.098 | Alcohol Consumption History | 2.422^b^ | 0.120 |
| Serum Creatinine | 1.900^a^ | 0.168 | Family History of Diabetes | 2.481^b^ | 0.115 |
| Total Protein | 0.108^a^ | 0.742 | History of Hypertension | 1.778^b^ | 0.182 |
| Total Cholesterol | 0.049^a^ | 0.826 | History of Chronic Heart Disease | 0.978^b^ | 0.323 |
| Low-Density Lipoprotein Cholesterol | 0.833^a^ | 0.361 | Hyperosmolar Hyperglycemic Syndrome | < 0.001^b^ | 0.990 |
| Hemoglobin | 1.876^d^ | 0.061 | Diabetic Foot | 0.386^b^ | 0.534 |
| Red Blood Cells | 0.776^a^ | 0.378 | Sleep Disorders | 3.352^b^ | 0.067 |
| White Blood Cells | 1.460^a^ | 0.227 | Oral Hypoglycemic Agents | 0.211^b^ | 0.646 |
| C-Reactive Protein | 1.104^a^ | 0.293 | Continuous Glucose Monitoring Use | 1.650^b^ | 0.199 |
| 25-Hydroxyvitamin D | 1.417^a^ | 0.234 | Urinary Protein | 3.789^c^ | 0.401 |
| Urinary Microalbumin | 1.509^a^ | 0.219 | Glutamic Acid Decarboxylase Antibodies (GADA) | 0.824^b^ | 0.364 |
| Potassium (K^+^) | 0.475^a^ | 0.490 | Zinc Transporter 8 Antibodies (ZnT8A) | 4.617^b^ | 0.099 |
| Sodium(Na^+^) | 0.251^a^ | 0.616 | Insulin Autoantibodies (IAA ) | 2.294^b^ | 0.130 |
| Calcium(Ca^2+^) | 1.516^a^ | 0.218 | Tyrosine Phosphatase Antibodies (IA-2A) | 1.160^b^ | 0.560 |
| Total Number of Complications | 9.704^b^ | 0.084 | Pancreatic Islet Cell Antibodies (ICA) | 1.115^b^ | 0.573 |

Table S5 Variance inflation factors of variables included in the final regression model

| **Variable** | **Tolerance** | **VIF** | **Variable** | **Tolerance** | **VIF** |
| --- | --- | --- | --- | --- | --- |
| Duration of alcohol consumption | 0.855 | 1.169 | Diabetic vascular complications | 0.785 | 1.274 |
| Inpatient mean blood glucose | 0.799 | 1.252 | Polypharmacy | 0.902 | 1.108 |
| Fasting C-peptide | 0.497 | 2.012 | Intravenous insulin injection | 0.763 | 1.311 |
| 2-hour C-peptide | 0.204 | 4.903 | Twice-daily subcutaneous insulin injection | 0.484 | 2.067 |
| Glomerular filtration rate | 0.503 | 1.989 | Once-daily subcutaneous insulin injection | 0.259 | 3.864 |
| Triglycerides | 0.861 | 1.161 | Single subcutaneous insulin injection plus intravenous insulin | 0.917 | 1.091 |
| High-density lipoprotein cholesterol | 0.937 | 1.067 | Multiple subcutaneous insulin injections (≥ 2 times) plus intravenous insulin | 0.926 | 1.079 |
| 25-Hydroxyvitamin D | 0.822 | 1.216 | Weekly insulin formulation | 0.918 | 1.089 |
| Employee medical insurance | 0.638 | 1.567 | Insulin pump use | 0.268 | 3.727 |
| Self-payment | 0.894 | 1.118 | Number of insulin types used-1 | 0.161 | 6.228 |
| History of hypoglycemia | 0.85 | 1.176 | Number of insulin types used-3 | 0.958 | 1.044 |
| Diabetic retinopathy | 0.855 | 1.169 | Number of insulin types used-4 | 0.875 | 1.143 |
| Diabetic nephropathy | 0.819 | 1.221 | Number of insulin types used-0 | 0.735 | 1.361 |
| Diabetic neuropathy | 0.814 | 1.228 | Educational level | 0.747 | 1.339 |
| Diabetic ketoacidosis | 0.658 | 1.520 | Diabetic ketosis | 0.673 | 1.485 |

Table S6 Sensitivity analysis evaluating synchronous coexistence between clinical indicators and prolonged autoimmune phenotype confirmation intervals across alternative model specifications

| **Variable** | **> 0 years (primary)** | | **OLS (log-continuous)** | | **≥ 2 years cutoff** | | **≥ 3 years cutoff** | |
| --- | --- | --- | --- | --- | --- | --- | --- | --- |
|  | **OR**  **(95% CI)** | ***p* value** | **Beta**  **(95% CI)** | ***p* value** | **OR**  **(95% CI)** | ***p* value** | **OR**  **(95% CI)** | ***p* value** |
| Intercept | 0.536  (0.201-1.428) | 0.2121 | 0.7347  (0.3020-1.1673) | 0.0009 | 0.478  (0.185-1.233) | 0.1268 | 0.406  (0.156-1.052) | 0.0635 |
| Educational level (high vs low) | 1.646  (1.111-2.437) | 0.0129 | 0.1772  (-0.0029-0.3573) | 0.0537 | 1.438  (0.981-2.107) | 0.0627 | 1.234  (0.839-1.813) | 0.2849 |
| Duration of alcohol consumption (log-years) | 1.162  (1.010-1.336) | 0.0353 | 0.1073  (0.0478-0.1668) | 0.0004 | 1.254  (1.097-1.433) | 0.0009 | 1.287  (1.133-1.462) | 0.0001 |
| Inpatient mean blood glucose (mmol/L) | 1.085  (1.001-1.177) | 0.0481 | 0.0136  (-0.0218-0.0490) | 0.4518 | 1.049  (0.972-1.133) | 0.2207 | 1.016  (0.942-1.096) | 0.6777 |
| 2-hour C-peptide (ng/mL) | 0.742  (0.653-0.844) | < 0.0001 | -0.0990  (-0.1442--0.0537) | < 0.0001 | 0.765  (0.673-0.870) | < 0.0001 | 0.811  (0.712-0.923) | 0.0016 |
| Diabetic retinopathy (yes vs no) | 2.289  (1.359-3.856) | 0.0019 | 0.4656  (0.2573-0.6740) | < 0.0001 | 1.856  (1.164-2.961) | 0.0094 | 2.015  (1.293-3.139) | 0.0020 |
| Diabetic ketosis (yes vs no) | 0.403  (0.273-0.595) | < 0.0001 | -0.3792  (-0.5526--0.2059) | < 0.0001 | 0.481  (0.331-0.699) | 0.0001 | 0.543  (0.373-0.790) | 0.0014 |
| Polypharmacy (yes vs no) | 2.221  (1.367-3.609) | 0.0013 | 0.3337  (0.1071-0.5603) | 0.0040 | 2.222  (1.368-3.611) | 0.0013 | 1.924  (1.159-3.195) | 0.0114 |

Notes: All models include the same seven independent variables (N = 578). All models significant overall (*p* < 0.001). Direction of association 100% consistent across all four specifications.
